# The *CYP19* RS4646 Polymorphism IS Related to the Prognosis of Stage I–II and Operable Stage III Breast Cancer

**DOI:** 10.1371/journal.pone.0121535

**Published:** 2015-03-20

**Authors:** Xiying Shao, Yong Guo, Xiaohong Xu, Yabing Zheng, Jiwen Wang, Zhanhong Chen, Jian Huang, Ping Huang, Jufen Cai, Xiaojia Wang

**Affiliations:** 1 Department of Medical Oncology, The Affiliated Zhejiang Cancer Hospital of Zhejiang Chinese Medical University, Hangzhou, Zhejiang Province, China; 2 Department of Oncology, The First Affiliated Hospital of Zhejiang Traditional Chinese Medical University, Hangzhou, Zhejiang Province, China; 3 Clinical laboratory, The Affiliated Zhejiang Cancer Hospital of Zhejiang Chinese Medical University, Hangzhou, Zhejiang Province, China; 4 Department of Oncology, Zhejiang Cancer Hospital, Hangzhou, Zhejiang Province, China; Central South University, CHINA

## Abstract

**Purpose:**

Aromatase, encoded by the *CYP19* gene, catalyzes the final step of the conversion of androgens to estrogens. Given the critical role of *CYP19* in estrogen synthesis, the potential influence of *CYP19* rs4646 polymorphism on breast cancer survival, deserves further study.

**Methods:**

Genotyping for *CYP19* rs4646 variants was performed on 406 Chinese women with stage I–II and operable stage III breast cancer. Associations were evaluated between *CYP19* rs4646 genotypes and disease-free survival (DFS).

**Results:**

In premenopausal patients, women who are homozygous for the minor allele (AA) have a longer DFS compared with those carrying the major allele (CC or AC) (87 months versus 48.7 months; Hazard ratio (HR) = 0.56, 95 % CI = 0.318-0.985, *P* = 0.041). These differences were further demonstrated by a multivariate analysis (HR = 0.456, 95 % CI = 0.249-0.836, *P* = 0.011). Conversely, the same variant (AA) was estimated to be associated with a poorer DFS in postmenopausal women (AA versus AC or CC: 13.7 months versus 56.3 months; HR = 2.758, 95 % CI = 1.432-5.313, *P* = 0.002). Furthermore, the differences were confirmed by the COX proportional hazards model (HR = 2.983, 95% CI =1.494-5.955, *P* = 0.002).

**Conclusions:**

The present study indicates that *CYP19* rs4646 polymorphism is related to DFS in early breast cancer and that the prognosis index of the homozygous for the minor allele (AA) may depend on menopause status. The findings are novel, if confirmed, rs4646 genotypes may provide useful information for routine management in breast cancer.

## Introduction

In last three decades, breast cancer have increased gradually worldwide [1]. It has been reported that in 2012, up to 226,870 women were diagnosed with breast cancer, and approximately three million people are estimated to be living with a history of breast cancer in the United States [2]. To our knowledge, estrogen is involved both in the development of the mammary gland, as well as in the pathogenesis and progression of breast cancer [3]. Aromatase, encoded by *CYP19* gene, catalyzes the final step of the conversion from androgens to estrogens [4–6]. In premenopausal patients, estrogen is mainly generated by the ovary, with a small fraction being produced by aromatization of adrenal and ovarian androgen in extragonadal tissue. Whereas, in postmenopausal women, aromatization of androgen from extragonadal tissue becomes the main source of estrogen since the ovary ceases to function [6–8].

Previous studies have demonstrated that polymorphisms in hormone-related genes were associated with clinical outcome in breast cancer [9]. In a cohort of stages I-II and operable stage III breast cancer patients, it has been estimated that hormone receptor-(HR-) positive premenopausal patients carrying the long allele of the *CYP19* TTTA polymorphism have a significantly longer disease-free survival (DFS) and overall survival (OS) than those without the long allele [10]. It has been suggested that in postmenopausal metastatic breast cancer (MBC) women with letrozole therapy, time to progression (TTP) was significantly prolonged in patients with the T allele of rs4646 compared with those carrying homozygotes for the wild-type variant (GG) [11]. Additionally, a study including 272 MBC women with anastrozole administration revealed that the rare allele of rs4646 was significantly associated with improved TTP as well as longer OS [12]. The other study, however, the same variants seemed to be correlated with a poorer benefit from letrozole therapy when evaluated in the neoadjuvant setting [13].

Given the critical role of *CYP19* gene in estrogen synthesis, the potential impact of *CYP19* genetic variants on survival, and hence management, deserves further study. In this prospective study, we performed a genetic analysis of *CYP19* polymorphisms in a cohort of 406 Chinese women with early breast cancer, and explored its clinical significance.

## Patients and Methods

### Study cohort and sources of information

Eligible women with stage I–II and operable stage III breast cancer were included between January 1, 2004 and June 30, 2010 in Zhejiang Cancer Hospital. Pathologic diagnosis was performed at the Department of Pathology, Zhejiang Cancer Hospital. A 2 mL blood sample was drawn and stored in polypropylene cryotubes at −80°C. All patients were provided written informed consent according to guidelines of the ethics committee of Zhejiang Cancer Hospital. This study was approved by the Review Board of Zhejiang Cancer Hospital.

### DNA preparation and genotyping

Genomic DNA was extracted from whole blood by the AxyPrep Blood Genomic DNA Miniprep kit (Axygen Biosciences, Union City, CA). Genotyping was performed with the SEQUENOM MassARRAY matrix-assisted laser desorption/ionization-time of flight mass spectrometry platform (Sequenom, San Diego, CA)[14]. Primers (5’-TCTCTTGTAGCCTGGTTCTC-3’and5’-GTGACAACCCATAGGAGGTA-3’) for polymerase chain reaction and single base extension were designed through the Assay Designer’s software version 3.0 (Sequenom) and synthesized by Sangon Biotech (Shanghai, China).

Purified primer extension reaction products were spotted onto a 384-well spectroCHIP with the MassARRAY Nanodispenser and determined by the matrix-assisted laser desorptionization time-offlight mass spectrometer. Genotype analysis was performed in real time with MassARRAY RT software version 3.0.0.4 and analyzed through the MassARRAY Typer software version 3.4.

### Statistical analysis

Follow-up data available as of May 30, 2014, were analyzed. DFS was measured from the date of the original surgery for breast cancer to the date of locoregional or distant recurrence or death for any causes [15]. Survival was calculated using the Kaplan- Meier method. Differences in survival were compared by the log-rank test.

The hazard ratio (HR) and the corresponding 95% confidence interval (CI) for each variable were estimated by Cox regression analyses. The Chi-square test and Fisher’s exact test were applied to compare differences between genetic polymorphisms and clinicopathologic parameters. The multivariate-adjusted HR of progression associated with the individual genotypes was assessed for the groups after adjusting for tumor size, lymph nodes involved, ER and PR status, HER-2 status, Body Mass Index (BMI), chemotherapy, adjuvant hormone therapy and radiotherapy. All statistical calculations were performed with SPSS 17.0 for Windows (SPSS Inc, Chicago, IL). Two-sided values less than 0.05 were considered statistically significant.

Deviation from Hardy–Weinberg equilibrium (HWE) was analyzed by Pearson's chi-squared test by means of the Finetti program [16].

## Results

### Clinicopathologic features and genetic polymorphism of *CYP19*

The median age was 45 years (range, 20–73 years); 294 were premenopausal and 112 were postmenopausal. Detailed information for the clinical outcome, patients characteristics were obtained. Briefly, all ER- and/or PR positive patients (n = 310) received tamoxifen (n = 255) or aromatase inhibitors (n = 55) as adjuvant hormonal therapy. 396 (97.5%) received chemotherapy including CAF (Cyclophosphamide, Doxorubicin and Fluoracil) or CEF (Cyclophosphamide, Epirubicin and Fluoracil) or AC (Doxorubicin, Cyclophosphamide) or TAC (Docetaxel, Doxorubicin and Cyclophosphamide), EC (Cyclophosphamide, Epirubicin) or AC (Doxorubicin, Cyclophosphamide) followed by Docetaxel or weekly Paclitaxel, CAF (Cyclophosphamide, Doxorubicin and Fluoracil) or FEC (Fluoracil, Epirubicin and Cyclophosphamide) followed by Docetaxel or weekly Paclitaxel treatment and others, 10 (2.5%) remained unknown. HER-2 positive women received Trastuzumab treatment. 203 (50.0%) received radiotherapy, 203 (50.0%) with no radiation.

Totally, there were 210 patients with CC genotype, 160 with AC variant, and 36 with AA genotype. Genotype frequencies observed in our patient cohort were consistent with Hardy–Weinberg equilibrium (*P* >0.05, data not shown). There were no significant differences between *CYP19* genotypes and patients features (Table 1).

**Table 1:** Correlation of *CYP19* polymorphism (CC vs AC vs AA) to clinical characteristics.

Characteristics	CCn (%)	ACn (%)	AAn (%)	n	*P* ^1^
Menopausal status					
Premenopausal	149 (71.0)	121 (75.6)	24 (66.7)	294	0.439
postmenopausal	61 (29.0)	39 (24.4)	12 (33.3)	112	
Tumor size (cm)					
≤ 2	68 (32.4)	54 (33.8)	12 (33.3)	134	0.925
>2–5	111 (52.9)	84 (52.5)	20 (55.6)	215	
>5	13 (6.2)	12 (7.5)	4 (11.1)	29	
Unknown	18 (8.6)	10 (6.3)	0	28	
Lymph node status					
0	59 (28.1)	49 (30.6)	9 (25.0)	117	0.512
1–3	77 (36.7)	45 (28.1)	10 (27.8)	132	
4–9	37 (17.6)	36 (22.5)	10 (27.8)	83	
>9	32 (15.2)	28 (17.5)	7 (19.4)	67	
Unknown	5 (2.4)	2 (1.3)	0	7	
TNM Stage					
I-II	120 (57.1)	84 (52.5)	16 (44.4)	220	0.081
III	69 (32.9)	64 (40.0)	20 (55.6)	153	
Unknown	21 (10.0)	12 (7.5)	0	33	
Hormone receptor status					
ER+PR+	114 (54.3)	88 (55.0)	23 (63.9)	225	0.596
ER+PR-	34 (16.2)	20 (12.5)	4 (11.1)	58	
ER-PR+	17 (8.1)	9 (5.6)	1 (2.8)	27	
ER-PR-	41 (19.5)	40 (25.0)	8 (22.2)	89	
Unknown	4 (1.9)	3 (1.9)	0	7	
HER-2 status					
Negative	64 (30.5)	50 (31.3)	10 (27.8)	124	0.163
+	40 (19.0)	46 (28.8)	11 (30.6)	97	
++	46 (21.9)	20 (12.5)	8 (22.2)	74	
+++	47 (22.4)	37 (23.1)	7 (19.4)	91	
Unknown	13 (6.2)	7 (4.4)	0	20	
HER-2 status					
Negative	119 (56.7)	103 (64.4)	24 (66.7)	246	0.646
Positive	56 (26.7)	41 (25.6)	8 (22.2)	105	
Unknown	35 (16.7)	16 (10.0)	4 (11.1)	55	
BMI					
<24	125 (59.5)	84 (52.5)	23 (63.9)	232	0.372
≥ 24	85 (40.5)	73 (45.6)	13 (36.1)	171	
Unknown	0	3 (1.9)	0	3	

^1^Two-sided χ^2^ test.

When the study patients were clustered into two groups, one with the CC or AC genotypes and the other carrying AA variant, the polymorphisms were not associated with clinicopathologic features (Table 2). Similarly, there was no relationship between genetic polymorphism and patients characteristics while subgrouped into two cohorts, one with CC genotype and the other carrying AC or AA variants (Table 3).

**Table 2:** Correlation of *CYP19* variants (CC vs AC + AA) to clinical characteristics.

Characteristics	CCn (%)	AC + AAn (%)	n	*P* ^1^
Menopausal status				
premenopausal	149 (71.0)	145 (74.0)	294	0.495
postmenopausal	61 (29.0)	51 (26.0)	112	
Tumor size(cm)				
≤ 2	68 (32.4)	66 (33.7)	134	0.789
>2–5	111 (52.9)	104 (53.1)	215	
>5	13 (6.2)	16 (8.2)	29	
Unknown	18 (8.6)	10 (5.1)	28	
Lymph node status				
0	59 (28.1)	58 (29.6)	117	0.214
1–3	77 (36.7)	55 (28.1)	132	
4–9	37 (17.6)	46 (23.5)	83	
>9	32 (15.2)	35 (17.9)	67	
Unknown	5 (2.4)	2 (1.0)	7	
TNM Stage				
I-II	120 (57.1)	100 (51.0)	220	0.073
III	69 (32.9)	84 (42.9)	153	
Unknown	21 (10.0)	12 (6.1)	33	
Hormone receptor status				
ER+PR+	114 (54.3)	111 (56.6)	225	0.295
ER+PR-	34 (16.2)	24 (12.2)	58	
ER-PR+	17 (8.1)	10 (5.1)	27	
ER-PR-	41 (19.5)	48 (24.5)	89	
Unknown	4 (1.9)	3 (1.5)	7	
HER-2 status				
Negative	64 (30.5)	60 (30.6)	124	0.060
+	40 (19.0)	57 (29.1)	97	
++	46 (21.9)	28 (14.3)	74	
+++	47 (22.4)	44 (22.4)	91	
Unknown	13 (6.2)	7 (3.6)	20	
HER-2 status				
Negative	119 (56.7)	127 (64.8)	246	0.395
Positive	56 (26.7)	49 (25.0)	105	
Unknown	35 (16.7)	20 (10.2)	55	
BMI				
<24	125 (59.5)	107 (54.6)	232	0.407
≥ 24	85 (40.5)	86 (43.9)	171	
Unknown	0	3 (1.5)	3	

^1^Two-sided χ^2^ test.

**Table 3:** Relationship of *CYP19* genotypes (CC + AC vs AA) with clinical characteristics.

Characteristics	CC + ACn (%)	AAn (%)	n	*P* ^1^
Menopausal status				
Premenopausal	270 (73.0)	24 (66.7)	294	0.419
Postmenopausal	100 (27.0)	12 (33.3)	112	
Tumor size (cm)				
≤ 2	122 (33.0)	12 (33.3)	134	0.713
>2–5	195 (52.7)	20 (55.6)	215	
>5	25 (6.8)	4 (11.1)	29	
Unknown	28 (7.6)	0	28	
Lymph node status				
0	108 (29.2)	9 (25.0)	117	0.641
1–3	122 (33.0)	10 (27.8)	132	
4–9	73 (19.7)	10 (27.8)	83	
>9	60 (16.2)	7 (19.4)	67	
Unknown	7 (1.9)	0	7	
TNM Stage				
I-II	204 (55.1)	16 (44.4)	220	0.062
III	133 (35.9)	20 (55.6)	153	
Unknown	33 (8.9)	0	33	
Hormone receptor status				
ER+PR+	202 (54.6)	23 (63.9)	225	0.649
ER+PR-	54 (14.6)	4 (11.1)	58	
ER-PR+	26 (7.0)	1 (2.8)	27	
ER-PR-	81 (21.9)	8 (22.2)	89	
Unknown	7 (1.9)	0	7	
HER-2 status				
Negative	114 (30.8)	10 (27.8)	124	0.758
+	86 (23.2)	11 (30.6)	97	
++	66 (17.8)	8 (22.2)	74	
+++	84 (22.7)	7 (19.4)	91	
Unknown	20 (5.4)	0	20	
HER-2 status				
Negative	222 (60.0)	24 (66.7)	246	0.524
Positive	97 (26.2)	8 (22.2)	105	
Unknown	51 (13.8)	4 (11.1)	55	
BMI				
<24	209 (56.5)	23 (63.9)	232	0.421
≥ 24	158 (42.7)	13 (36.1)	171	
Unknown	3 (0.8)	0	3	

^1^ Two-sided χ^2^ test.

### *CYP19* polymorphisms and DFS

**Fig 1:**
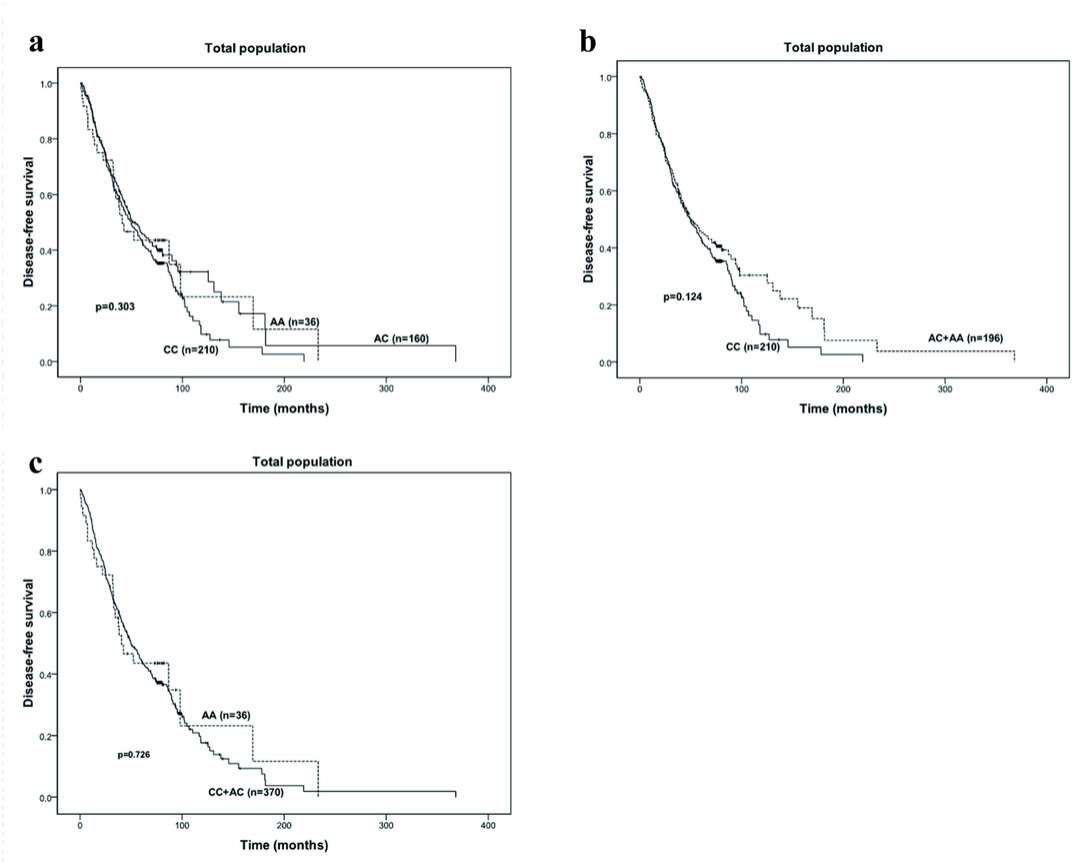
Survival curves for the total patients. a. Disease-free survival of the patients grouped according to *CYP19* rs4646 genotypes (CC vs AC vs AA). b. Disease-free survival of the patients grouped by *CYP19* rs4646 genotypes (CC vs AC + AA). c. Disease-free survival of the patients subgrouped according to *CYP19* rs4646 genotypes (AC+CC vs AA).

The median follow-up time was 96 months (range 47–125 months). Overall, there was no significant difference in DFS among patients with these three genotypes (CC versus AC versus AA: 49.7 months versus 51.0 months versus 40.8 months, *P* = 0.303) (Fig. 1a). Moreover, no relationship was observed between the patients with wild-type genotype (CC) and those carrying AC or AA variants (49.7 months versus 49.7 months; *P* = 0.124) (Fig. 1b), between CC or AC genotype and AA variant (49.7 months versus 40.8 months; *P* = 0.726) (Fig. 1c).

**Fig 2:**
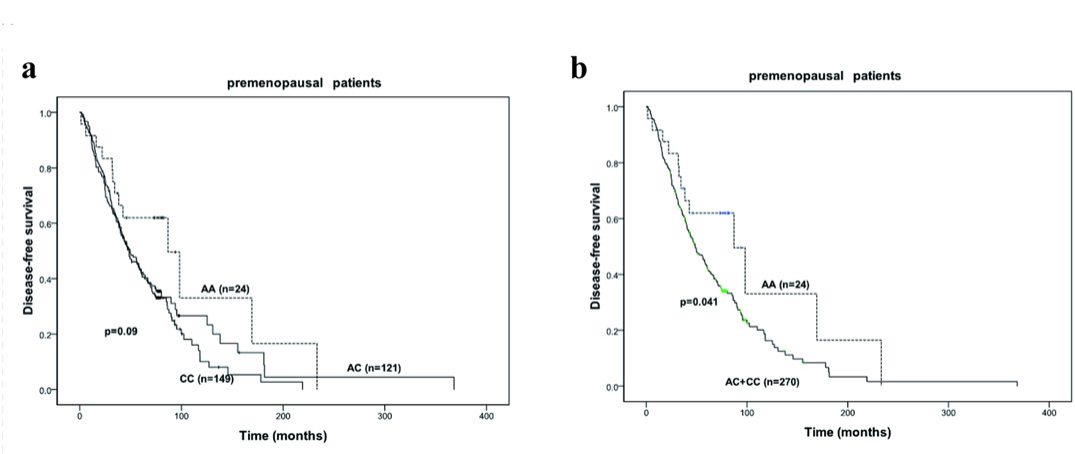
Survival curves for the premenopausal patients. a. Disease-free survival of the premenopausal women stratified by *CYP19* rs4646 genotypes (CC vs AC vs AA). b. Disease-free survival of the premenopausal women grouped according to *CYP19* rs4646 genotypes (AA vs AC + CC).

In premenopausal women, AA genotype tended to be in relation with longer DFS (AA versus AC versus CC: 87.0 months versus 48.4 months versus 49.7 months, *P* = 0.090) (Fig. 2a). When the patients were divided into two groups, one with AA variant and the other carrying CC or AC genotypes, AA genotype was significantly associated with prolonged DFS (87 months versus 48.7 months; HR = 0.56, 95% CI = 0.318–0.985, *P* = 0.041) (Fig. 2b). Furthermore, being adjusted by positive lymph nodes, tumor size >5 cm, negative hormone receptor status, HER-2-postive status, chemotherapy, radiation and hormone therapy in multivariate analyses, AA genotype remained an independent prognostic factor for DFS (HR = 0.456, 95% CI = 0.249–0.836, *P* = 0.011) (Table 4).

**Table 4:** Multivariate analysis of prognostic factors and DFS for premenopausal patients.

Characteristics	HR (95% CI)	*P* ^1^
*CYP19* polymorphism		
AA versus AC + CC	0.560 (0.318–0.985)	**0.041**
Tumor size		
>5 versus ≤ 5 cm	1.035 (0.975–1.099)	0.261
Lymph nodes		
Positive versus negative	1.060 (0.951–1.181)	0.294
ER		
Positive versus negative	0.938 (0.825–1.377)	0.625
PR		
Positive versus negative	0.812 (0.610–1.081)	0.154
HER-2		
Negative versus positive	0.943 (0.891–0.997)	**0.039**
BMI		
>24 versus ≤ 24	1.163 (0.944–1.433)	0.157
Adjuvant chemotherapy		
Yes versus No	0.863 (0.768–0.969)	**0.013**
Adjuvant radiotherapy		
Yes versus No	0.866 (0.709–1.059)	0.161
Adjuvant hormone therapy		
Yes versus No	0.909 (0.698–1.060)	0.299

Note: HR, hazard ratio; CI, confidence interval.

^1^Data were estimated by Cox regression analyses with adjustment for tumor size, lymph nodes involved, ER and PR status, HER-2 status, Body Mass Index (BMI), chemotherapy, adjuvant hormone therapy and radiotherapy.

**Fig 3:**
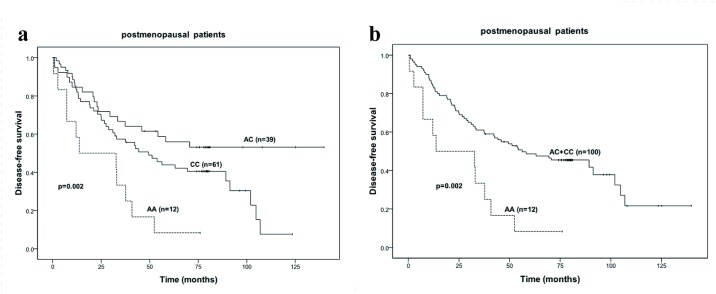
Survival curves for the postmenopausal patients. a. Disease-free survival of the postmenopausal women grouped by *CYP19* rs4646 genotypes (CC vs AC vs AA). b. Disease-free survival of the postmenopausal women stratified by *CYP19* rs4646 genotypes (AA vs AC + CC).

In postmenopausal women, AA genotype was evident to be in relation with shorter DFS (AA versus AC versus CC: 13.7 months versus not reached versus 49.4 months, *P* = 0.002) (Fig. 3a). Moreover, there was significant difference in DFS between the patients with AA variant and those carrying CC or AC genotype (AA versus AC or CC: 13.7 months versus 56.3 months; HR = 2.758, 95% CI = 1.432–5.313, *P* = 0.002) (Fig. 3b). In the Cox proportional hazards model, after adjusting for the patients features, AA variant was explored to be an independent prognostic factor for DFS (HR = 2.983, 95% CI = 1.494–5.955, *P* = 0.002) (Table 5).

**Table 5:** Multivariate analysis of prognostic factors and DFS for postmenopausal patients.

Characteristics	HR (95% CI)	*P* ^1^
*CYP19* polymorphism		
AA versus AC + CC	2.983 (1.494–5.955)	**0.002**
Tumor size		
>5 versus ≤5 cm	1.055 (0.855–1.302)	0.617
Lymph nodes		
Positive versus negative	1.330 (1.029–1.717)	**0.029**
ER		
Positive versus negative	0.939 (0.499–1.764)	0.844
PR		
Positive versus negative	0.669 (0.385–1.162)	0.153
HER-2		
Negative versus positive	0.971 (0.879–1.073)	0.560
BMI		
>24 versus ≤24	1.001 (0.815–1.230)	0.992
Adjuvant chemotherapy		
Yes versus No	0.646 (0.370–1.092)	0.102
Adjuvant radiotherapy		
Yes versus No	0.886 (0.526–1.495)	0.650
Adjuvant hormone therapy		
Yes versus No	0.880 (0.627–1.236)	0.462

Note: HR, hazard ratio; CI, confidence interval.

^1^Data were estimated by Cox regression analyses with adjustment for tumor size, lymph nodes involved, ER and PR status, HER-2 status, Body Mass Index (BMI), chemotherapy, adjuvant hormone therapy and radiotherapy.

## Discussion

In the present study, we demonstrated that, premenopausal women with homozygous for the minor allele of *CYP19* rs4646 had significantly longer DFS than those carrying the major allele, however, the same homozygous variant was estimated to be associated with poorer DFS among the postmenopausal women. These differences were further confirmed by multivariate analysis. The findings are biologically plausible, given the crucial role of aromatase in estrogen synthesis, its identified impact on tumor growth and progression, and the potential functional significance of *CYP19* genetic polymorphisms.

Population-based studies of *CYP19* polymorphisms have generated inconsistent results with regard to their potential association with clinical outcome. It has been suggested that rare T allele of rs4646 was correlated with prolonged TTP in postmenopausal MBC women with letrozole therapy. Additionally, the frequency of the variant allele was significantly higher in the responder group (61% vs 40%) [11]. Likewise, Liu et al [12] estimated that this minor allele of rs4646 was significantly linked with longer TTP and OS when assessed in MBC women with anastrozole administration. The other study, conversely, the same variant was revealed to be in relation with shorter progression-free survival (PFS) in the neoadjuvant setting. Besides, the genotypic variants of rs4646 were more frequently represented in the nonresponder cohort (48% vs 26%) [13]. Similarly, the data in 296 early breast cancer patients indicated that the combined high risk A/A + A/C alleles of *CYP19* polymorphism rs4646 were significantly related to poorer distant disease-free survival (DDFS) and marginally associated with shorter DFS and OS [17]. However, other studies did not observe any significant differences between the rs4646 polymorphisms with clinical outcome [18–20].

Given the critical role of hormone in the pathogenesis and progression of breast cancer, the circulating estrogen levels may have negative impact on survival in women with breast cancer [3]. Lønning et al. [21]showed that circulating estrogen levels were significantly associated with poorer DFS in postmenopausal patients. In a case–control cohort study, Rock et al. [22] indicated that total estradiol, bioavailable estradiol, and free estradiol circulating concentrations were correlated with risk of recurrence. Besides, it has been suggested that *CYP19* polymorphisms were significantly associated with hormone levels [23–25]. Haiman et al. [26]demonstrated that patients with the 8-repeat allele of the TTTA polymorphism have higher estrogen levels than those carrying the 7-repeat allele. The analysis in five large prospective cohorts showed that rs727479 and rs749292 were significantly related to higher estradiol and estrone levels [4]. More recently, some data indicated that the rs4646 may be linked with circulating hormone levels in postmenopausal breast cancer [11,13]. Interestingly, rs1065779 of *CYP19* has been estimated to have impact on transcription or expression of aromatase [27].

Elevated levels of aromatase expression have been observed in breast tumors relative to normal breast tissue [28,29]. Meanwhile, some other analysis have indicated a significant association between aromatase and estrogen-related receptor mRNA expression in isolated tumor cells [30]. A number of studies showed that *CYP19* polymorphisms were relevant to greater aromatase activity. Kristensen et al [7]showed that a higher number of TTTA repeats of *CYP19* was associated with greater aromatase activity. Likewise, another study revealed that the same genotype was in relation with aromatase activity [31]. In addition, the data in anastrozole neoadjuvant setting indicated that rs6493497 and rs7176005 were correlated with much more decrease in aromatase activity [32]. Meanwhile, a population-based and in vitro study carried out by Ma et al [33] revealed reductions in the functional activity of aromatase for four phenotypes with non-synonymous changes. The authors observed that protein levels or aromatase activity decreased sharply for the Thr364 and a slight reduction in Cys264 allozyme activity. The mechanism by which non-synonymous SNPs interfere with the enzymatic activity is a consequence of an alteration in the protein level [33]. The data available is not sufficient to confirm that rs4646 is an activating polymorphism, but the previous data indicate that it could be related to an advantage in the protein structure which makes it more active [11].

On the basis of the relationship between *CYP19* polymorphisms and the estrogen level as well as aromatase activity, mentioned above, we speculate that, premenopausal patients carrying AA genotype may harbor higher estrogen levels, and it is likely that the majority of the premenopausal women included in our study had menopause due to adjuvant therapies, and what’s more, the treatment-induced decrease in levels of circulating estrogen may be more remarkable among patients with AA variant than those carrying AC or CC genotype. Therefore, adjuvant therapy might be more effective in premenopausal patients with AA variant. However, the changes in estrogen levels caused by adjuvant therapy is not so great between postmenopausal patients with AA genotype and those carrying AC or CC variant, and thus, women who are homozygous for the minor allele (AA) have a poorer DFS compared with those carrying the major allele (CC or AC).

There are some limitations to the present study. Firstly, the tested was limited to rs4646 of which the influence on breast cancer is controversial, because of the exploratory nature of the current study. Secondly, the genetic polymorphisms may have impact on phenotypic outcome through altering DNA binding sites [34,35], mRNA stabilization, splicing, folding [36–38] and regulation of the transcription and the posttranslational modification. Therefore, clarifying the molecular mechanisms of the effect of *CYP19* rs4646 polymorphism, such as transcription influence, mRNA stabilization, post-translational regulation of aromatase levels and aromatase activity is needed.

In summary, the present study indicates that the homozygous variant AA exerts a better effect in DFS in premenopausal patients, but a worse impact on DFS in postmenopausal women, which implies premenopausal women with AC or CC genotypes and postmenopausal women with AA genotype might receive more active treatment and more frequently follow-up beyond routine clinical management. These findings are novel, further validation in a larger independent cohort of early breast cancer patients is warranted.
